# Management of multiple sclerosis fatigue in the digital age: from assessment to treatment

**DOI:** 10.3389/fnins.2023.1231321

**Published:** 2023-10-05

**Authors:** Chiara Pinarello, Julia Elmers, Hernán Inojosa, Christian Beste, Tjalf Ziemssen

**Affiliations:** ^1^Center of Clinical Neuroscience, Department of Neurology, University Hospital Carl Gustav Carus, Technical University of Dresden, Dresden, Germany; ^2^Cognitive Neurophysiology, Department of Child and Adolescent Psychiatry, Faculty of Medicine, Technical University of Dresden, Dresden, Germany

**Keywords:** multiple sclerosis, fatigue, digital health, virtual reality, digital biomarkers, wearable devices, disease management, apps

## Abstract

Fatigue is one of the most disabling symptoms of Multiple Sclerosis (MS), affecting more than 80% of patients over the disease course. Nevertheless, it has a multi-faceted and complex nature, making its diagnosis, evaluation, and treatment extremely challenging in clinical practice. In the last years, digital supporting tools have emerged to support the care of people with MS. These include not only smartphone or table-based apps, but also wearable devices or novel techniques such as virtual reality. Furthermore, an additional effective and cost-efficient tool for the therapeutic management of people with fatigue is becoming increasingly available. Virtual reality and e-Health are viable and modern tools to both assess and treat fatigue, with a variety of applications and adaptability to patient needs and disability levels. Most importantly, they can be employed in the patient's home setting and can not only bridge clinic visits but also be complementary to the monitoring and treatment means for those MS patients who live far away from healthcare structures. In this narrative review, we discuss the current knowledge and future perspectives in the digital management of fatigue in MS. These may also serve as sources for research of novel digital biomarkers in the identification of disease activity and progression.

## 1. Introduction

Multiple Sclerosis (MS) is a chronic inflammatory autoimmune disease of the central nervous system (CNS). The multifactorial pathophysiology of MS leads to demyelination of axons in the CNS affecting every neurological functional system (National Multiple Sclerosis Society, 1991). Consequently, the clinical presentation is extremely heterogeneous including, among several other symptoms, fatigue. Fatigue is one of the most disabling complaints of MS patients, affecting up to 80% over the disease course (Chalah et al., 2015; Rooney et al., 2019; Palotai and Guttmann, 2020; Oliva Ramirez et al., 2021). Patients across every disease phenotype may be affected already in the early stages of the disease, with high interpersonal variability in the quality, frequency, and severity of fatigue (Filippi and Rocca, 2020).

However, management of this symptom is often limited in clinical and scientific practice, even considering its relevance for the care of patients. Therapy success is directly related to a correct assessment and monitoring of fatigue. Objective measures for assessing fatigue beyond the anamnesis have been, nonetheless lacking until the most recent years, as novel outcome measures and tools have emerged (Voigt et al., 2021; Ziemssen and Haase, 2021). These include patient-reported outcome measures as well as several digital tools for the assessment of the disease at several levels. Similarly, effective symptomatic treatment is currently relatively limited as few evidence-based options are available. As pharmacotherapy in the treatment of fatigue is not established, cognitive training and neuro-rehabilitation seem to play a key role in disease management (Sailer et al., 2023).

In this review, we discuss a clinical and digital perspective regarding assessing and managing fatigue. Moreover, we explore the state of the art in the technological field of treating this fundamental symptom from current definitions to diagnosis and discuss procedures used in daily practice (clinical evaluation, EDSS, cognitive and neuropsychological tests). We focused on the critical points and limitations of current procedures and potential improvements through digital tools. In this context, we highlight current and future uses of newer resources such as apps and wearable devices for patient awareness and active management of the disease. The use of digital applications may additionally offer a resource beyond assessment but also for the treatment of patients with fatigue.

## 2. Current state-of-the-art in fatigue in MS

### 2.1. Definition and diagnosis

A clear and strict definition of fatigue is fundamental for proper assessment and treatment taking advantage of digital tools. This is, in contrast to several other MS-related symptoms, frequently not clear or standard in clinical practice as a broad spectrum of complaints or features is reported (Mills and Young, 2008). Additionally, several classifications and considerations may apply in the assessment of this symptom.

Mills and Young (2008) defined fatigue in an MS population as a “reversible, motor and cognitive impairment with reduced motivation and desire to rest”.

A broad spectrum of features is described by patients with fatigue, including motor or cognitive dysfunction, lack of motivation, and rest complaints or behavioral responses (e.g., daytime resting, activity avoidance) (Mills and Young, 2008).

When discussing fatigue, a distinction between perceived fatigue and performance fatigability, the two facets contributing to its definition, is of capital importance (Enoka and Duchateau, 2016). In the framework of a neurologic disease such as MS, perceived fatigue represents an individual's perception of tiredness and thus disparity between the energy exerted in an activity and the actual outcome of it. In other words, the energy exerted in overcoming a task is greater than the one the task normally requires (Kluger et al., 2013; Enoka and Duchateau, 2016). Performance fatigability is the actual and measurable physical and cognitive drop in performance due to this state of exhaustion. Consequently, it is important to distinguish between the perception of the patient (perceived fatigue) and the tangible and measurable decrease in performance (performance fatigability). In MS, perceived fatigue can be reported by the patient through a self-assessment-designed questionnaire either at resting state or when carrying out an activity, whereas performance fatigability is preferably estimated during motor effort (e.g., walking long distances). Additionally, the latter can be measured in terms of change in task achievement over some time (Kluger et al., 2013; Enoka and Duchateau, 2016; Linnhoff and Heinze, 2019; Enoka et al., 2021). Additionally, cognitive fatigability (which is documented to decrease attention, processing speed, and memory) has to be differentiated from motor fatigability (which affects the ability to carry out physical tasks) (Enoka and Duchateau, 2016; Harrison et al., 2017).

Some authors also divide fatigue into state and trait fatigue, which is a further definition based on time and change: the former refers to a more acute situation, whereas trait fatigue belongs to a more chronic manifestation of fatigue (Kluger et al., 2013; Linnhoff and Heinze, 2019).

Although fatigue and fatigability essentially result from a central phenomenon (since MS is a disease of the central nervous system), there are some central al peripheral factors to take into consideration when exploring the phenomenology of fatigue. On the one end, central factors related to the pathophysiology of MS include damage to the cortical and subcortical areas, which then show further functional instability as the connectivity and therefore the performance is impaired.

The damage caused in the brain by MS is macro-structural (i.e., brain atrophy and volume loss) and microstructural (e.g., disrupted white matter integrity), involving precise areas of the nervous system (Jameen et al., 2019). In a vicious cycle, the interruption of the white matter (WM) tracts between the brain stem and the brain itself promotes the “neuro-immune reaction”, that is to say, the imbalance between the production of inflammatory cytokines and the inhibition of anti-inflammatory ones (Carandini et al., 2021).

**Figure 1:**
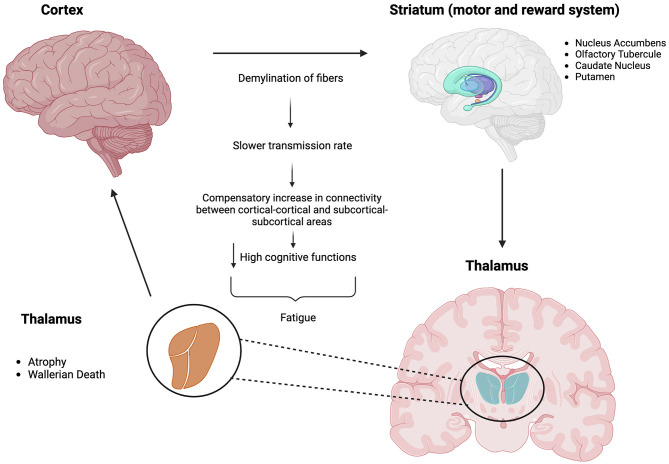
The cortico-striato-thalamo-cortical loop (CSTCL). This circuit involves the cortex, the basal ganglia (striatum, in turn consisting of nucleus accumbens, olfactory tubercule, caudate nucleus, and putamen), and the thalamus and ends up in the cortex. The fibers present in this circuit convey dopamine and glutamine among others. They control cognition and more especially decision-making, problem-solving, and planning. Created with BioRender.com.

As far as the specific pathophysiological mechanism of MS fatigue, the damage in the cortico-striato-thalamo-cortical loop (CSTCL, represented in Figure 1) seems to play a role in its onset at a central level. This loop conveys high cognitive functions like planning, problem-solving, and goal-setting. The demyelination of such fibers produces a slower transmission rate and a consequent difficulty in carrying on cognitively intense tasks (Jameen et al., 2019; Capone et al., 2020). Monoamines such as dopamine, serotonin, and noradrenaline are other keystones of CNS functional integrity, representing a bridge between the immune and the nervous system. In addition, the meso-cortico-limbic circuit is responsible for carrying dopamine from the ventral tegmental area to the nucleus accumbens, amygdala, hippocampus, and prefrontal cortex. This is also called “the reward pathway” for its importance in motivation and arousal, which lack in fatigued MS patients (Carandini et al., 2021).

On the other hand, peripheral factors involve local muscle activation (Kluger et al., 2013). Nevertheless, this distinction must not be taken as a standard definition, as the perception of fatigue and performance fatigability are intertwined and the physiological processes contributing to these conditions are complex (Enoka and Duchateau, 2016).

At later stages, a smoldering neurodegenerative process, partially driven by early inflammation, results in additional motor and cognitive deterioration (Giovannoni et al., 2022). Local neuro-axonal loss, Wallerian death, demyelination, and autoimmune reaction only partly account for the clinical and functional decline. Chronic inflammatory activity is decisive in determining disease progression and the pathophysiological base of fatigue (Giovannoni et al., 2022).

Finally, some factors responsible for secondary fatigue are depression, sleep deprivation, infections, pain, and adverse reactions to medications (Kluger et al., 2013; Khan et al., 2014; Adibi et al., 2022). Identifying and ruling out these concurring conditions is necessary for the differential diagnosis of primary MS and secondary fatigue (Kluger et al., 2013).

### 2.2. Impact of fatigue on quality of life

Fatigue is not only present in people with progressive MS but also in the early stages of the disease (Jameen et al., 2019; Rooney et al., 2019; Chang et al., 2023). Young patients with MS, at the peak of their productivity, may complain of this symptom, as up to 40–60% consider fatigue the most disabling MS symptom (Bakshi, 2003; Shah, 2015). Similarly to the MS epidemiology, a higher prevalence of fatigue is observed in women and patients with a higher disability or more active disease (Broch et al., 2021). The poignancy of fatigue as a pivotal symptom of MS is shown in recent literature as the patients regard it as a fundamental element when it comes to the therapy of choice. Tervonen et al. observed how they are more willing to endure an increased rate of relapses and accelerated progression in exchange for alleviating cognitive and physical fatigue. This was especially true when considering the ones who had received their diagnosis more than a decade before (Tervonen et al., 2023).

Fatigue may result in a significant economic burden related to high absence rates at work, unemployment, and the need for disability pensions (Oliva Ramirez et al., 2021). Frequently, it leads to a reduction in working hours and productivity, unemployment, or even early retirement (Braley and Chervin, 2010; Schiavolin et al., 2013; Mäcken et al., 2021). Fatigue may cause working MS patients to quit their jobs around 3 years after the diagnosis (García-Domínguez et al., 2019). Nevertheless, while fatigue has traditionally been considered a prognostic factor for unemployment, recent scientific literature has introduced a counterargument. In particular, emerging evidence highlights cognitive performance fatigability, with a particular emphasis on alertness, as a more potent predictor, in contrast to the self-reported fatigue that individuals with multiple sclerosis might convey through surveys and questionnaires (García-Domínguez et al., 2019; Dettmers et al., 2021; Oliva Ramirez et al., 2021).

Another consequence of fatigue in MS patients is social isolation, as the motivation to have social encounters or even simple conversations may be impaired. Moreover, daily activities such as housekeeping or self-hygiene might represent tremendous efforts. Lack of energy and motivation affects not only motor capability but also cognitive functions, making patients less confident and more dependent on caregivers in general (Penner et al., 2020; Battaglia et al., 2022). Quality of Life (QoL), which can be evaluated through scales like the MSQOL-54 (Multiple Sclerosis Quality of Life) (Janardhan and Bakshi, 2002), is therefore significantly affected by fatigue in MS patients. It is a self-reported questionnaire encompassing their health status. It includes both MS-specific and non-specific items and is a valid instrument to assess the impact of MS on the patient's daily routine (Vickrey et al., 1995). Fatigue has a significant impact on QoL along with the level of disability, as observed by Janardhan and Bakshi (2002). A more recently developed scale is the PROMIS fatigue 8a, a shorter version of the PROMIS scale, retrospectively measuring the specific impact of MS fatigue in 7 days. It is quick to administer, precise, and sensitive to subtle changes in fatigue level, which makes it another useful assessment tool in clinical practice (Kamudoni et al., 2022).

### 2.3. Traditional assessment of MS fatigue

In clinical care, assessment of fatigue is frequently limited to the patient's clinical history and the classical clinical examination (Sander et al., 2017; Gumus, 2018). In the setting of clinical trials, examinations may be more extensive although still limited, as specific scales or questionnaires have been established to quantify MS symptoms. Following, we describe the most used tools for assessing fatigue. Some of these measures are already taking advantage of digitalization and research advances, as digital devices aid the collection of data.

The EDSS is the most used scale for evaluating disability in MS patients. Through the clinical examination of eight domains (visual, brainstem, pyramidal, cerebellar, sensory, bowel and bladder, cerebral, and ambulatory) the clinician and therefore the clinical researcher can rate the patients' disability on a 1–10 scale. In particular, the Cerebral Functions domain provides an estimation of depression, cognitive dysfunction, and fatigue (Kurtzke, 1983a,b). The evaluation of fatigue is here limited to a single subdomain and rated through an extremely simplified method. A known limitation is the lack of responsiveness and sensitivity of the EDSS to detect disability changes, especially in domains beyond motor dysfunction (Inojosa et al., 2020). Moreover, the focus of the Cerebral Functions domain in the EDSS does not include an extensive analysis of fatigue and its correlates in the patient's life and activities of daily living (Cadavid et al., 2017; Schmidt and Jöstingmeyer, 2019; Dillenseger et al., 2021; Giovannoni et al., 2022).

Patient-reported outcomes (PROs) are evaluations of health status performed by patients themselves (El Gaafary, 2016). Through PROs, they provide an insight into their perception of a symptom (e.g., fatigue) (Bharadia et al., 2022) and therefore assure high test-retest reliability (Inojosa et al., 2020). In the last decade, PRO measures emerged as a relatively effective, simple, and reliable method to assess disability in MS. In the specific evaluation of fatigue, fatigue diaries and numerous scales for the clinical and neuropsychological examination are summarized in Table 1.

**Table 1:** Fatigue scales mostly in use.

**Name**	**Author**	**Date**	**Content**	**Scoring**	**References**
Fatigue Severity Scale (FSS)	Krupp et al.	1983	9 items: motivation, exercise, physical functioning, difficulty in carrying out tasks, work and private life impairment	7-points likert scale 1 = strongly disagree 7 = strongly agree Range: 9–63	Krupp, 1983a,b; Krupp et al., 1988; Schwartz et al., 1993
Modified Fatigue Impact Scale (MFIS)^*^	Fisk et al.	1994	21 items: physical (0–36), cognitive (0–40), psychosocial (0–8) subscale	5-points likert scale 0 = never 4 = almost always Range: 0–84	Fisk et al., 1994a,b
Fatigue Scale For Motor and Cognitive Functions (FSMC)^*^	Penner et al.	2005	20 items: motor (0-50) and cognitive (0-50) subscale	5-points likert scale: 1 = does not apply 5 = applies completely Range: 20-100	Penner et al., 2005, 2009
Neurological Fatigue Index (NFI-MS)^*^	Mills et al.	2010	23 items: physical subscale (8 items), cognitive subscale (4 items), diurnal sleep subscale (6 items), abnormal nocturnal sleep subscale (5 items)	4-point likert scale: 0 = strongly disagree 3 = strongly agree Range: 0–69 Another 10 points can be given in the summary section (range: 0–30)	Mills et al., 2010
Fatigue Assessment Inventory (FAI)^*^	Schwartz et al.	1993	29 items: severity of fatigue, impact on physical activity and daily life, interaction with rest, mood	7-points likert scale: 1 = disagree 7 = agree Range: 29–203	Schwartz et al., 1993
Fatigue Descriptive Scale (FDS)^*^	Iriarte et al.	1999	5 items: initiative (patient explains perception of fatigue), modality of onset, gravity, frequency, Unthoff's phenomenon	3-point likert scale Range: 0–17	Iriarte et al., 1999
Rochester Fatigue Diary (RFD)^*^	Schwid et al.	2002	Visual scale, fatigue level	100 mm visual analog scale, patient should mark his/her fatigue level every hour for 1 day 0 = maximal fatigue 100 = no fatigue	Schwid et al., 1987, 1999, 2002
PROMIS-Fatigue (MS)^*^	Cook et al.	2012	8 items: capacity to think clearly, enjoy life, tiredness, interference with social activities	5-points likert scale 1 = never 5 = always Range: 8–40	Cook et al., 2012; UWCORR, 2020
Multidimensional Fatigue Inventory (MFI)^*^	Smets et al.	1995	20 items: general, physical, cognitive, reduced motivation, reduced activity subscales	5-points likert scale 1 = true 5 = not true Range: 20–100	Smets et al., 1995
Fatigue Assessment Scale (FAS)^*^	Michielsen et al.	2003	10 items: fatigue intensity, onset, limitations, concentration	5-points likert scale: 1 = never 5 = always Range: 10–50	Michielsen et al., 2003

* RF (Shahid et al., 2012; Gumus, 2018; Rietberg and van Wegen, 2019).

The inclusion of extensive neuropsychological examinations is increasingly gaining importance in the quantification of fatigue and related cognitive manifestations, which are just partially rated in the EDSS (Strober et al., 2019). In the correct setting, neuropsychological tests include some of the most accurate predictors of outcomes related to occupation and the activities of daily living (ADL) in MS (Weber et al., 2019). One of the most established tests is the Symbol Digit Modalities Test (SDMT). The SDMT is a paper-based test in which patients have to pair numbers and symbols as fast as possible, referring to a legend present at the top of the sheet (Benedict et al., 2006). Although the SDMT is not specifically developed for the assessment of fatigue and it cannot replace an extensive neuropsychological assessment, data shows a tendency toward emerging fatigue during cognitively demanding and goal-oriented tasks (Sander et al., 2017; Sandry et al., 2021). Evaluations of fatigue and cognitive function are intrinsically related, and the neuropsychological examination seems to be a valuable resource to properly assess fatigue, cognitive impairment, and psychological-driven complaints. As previously mentioned, it is objective cognitive fatigability that is more predictive of lower employment outcomes in the future of the patients rather than the perception of fatigue. Cognitive fatigability is easily and quickly assessed through TAP (Test for Attention) alertness examinations. The goal of these is to test their sustained attention during tasks requiring processing visual stimuli as quickly as possible. This correlates accurately with fatigue levels and unemployment status after a 3-month rehabilitation (Zangemeister et al., 2020; Dettmers et al., 2021).

Overall, good management of fatigue in MS starts with accurate, continuous, and multimodal monitoring. This includes the PROs and objective data regarding acute symptoms, chronic impairments, and comorbidities (Voigt et al., 2023).

### 2.4. Traditional management

#### 2.4.1. Pharmacological treatment

An extensive discussion of pharmacological and non-pharmacological treatment of fatigue is beyond the scope of this paper. It is however well-known that therapeutic management of fatigue is extremely limited in MS. No consensus is available regarding pharmacological measures as no specific, evidence-based options are available.

The use of DMTs may improve fatigue symptoms, among other neurological deficits, as observational studies including the use of glatiramer acetate, interferon or natalizumab suggest an improvement. However, high-quality evidence including blinded, randomized, controlled trials is lacking (Metz, 2004; Svenningsson et al., 2013; Neuhaus et al., 2021).

Evidence is dispersed, fragmented, and contradictory (Pucci et al., 2007; Jensen et al., 2014), partially due to the not fully understood pathogenesis of fatigue. Several medications have been originally developed for other diseases and tested in MS (e.g., sleep disorders, influenza, Parkinson's, ADHD, etc...) (Zielińska-Nowak et al., 2020; Moss-Morris et al., 2021; Tarasiuk et al., 2022). Amantadin, 4-aminopyridine, and modafinil are, to date, the most often prescribed drugs, although their range of reliability has been low. They comprehend all off-label preparations, being, respectively an antiviral, an anti-spastic, and an anti-narcolepsy drug, and bearing numerous contraindications and side effects (e.g., heart, liver, and kidney damage as well as a potential teratogenic effect for amantadine) (Jensen et al., 2014; Picariello et al., 2022; Deutsche Multiple Sklerose Gesellschaft B e. V., 2023). A few other preparations have been tested to treat fatigue and they are summarized in Table 2.

**Table 2:** Most prescribed drugs against fatigue.

**Name**	**Mechanism**	**Effect**	Dose^*^	**Most common adverse reactions**	**References**
4-Aminoipyridine (4-AP)	Voltage-gated potassion channel inhibitor	Improves neurochemical conduction of demyelinated axons, increases release of neurotransmitters (dopaminergic effect)	10–40 mg/day	Paresthesia, restlessness, balance disorders, UTI, insomnia, dizziness, headache, nausea	Jensen et al., 2014; Zielińska-Nowak et al., 2020; Dietrich et al., 2021
Amantadine	Antiviral, immune-mediated, amphetamine-like activity	Improves cholinergic and dopaminergic transmission, non-specific CNS stimulant (approved by the FDA for Influenza and Parkinson's)	100–200 mg/day	Livido reticularis, insomnia, orthostatic hypotension, peripheral edema, headache, insomnia, dizziness	Krupp, 2003; Pucci et al., 2007; Braley and Chervin, 2010; Zielińska-Nowak et al., 2020; Cocco and Fadda, 2022; Tarasiuk et al., 2022
Modafinil	Non-amphetamine, CNS stimulant	Promotes wakefulness, probable sympathomimetic activity (used for narcolepsy or shift-work sleep disorders)	200–400 mg/day	Headache, anxiety, dizziness, nausea, hypertension, palpitations, insomnia	Braley and Chervin, 2010; Niepel et al., 2013; Zielińska-Nowak et al., 2020; Cocco and Fadda, 2022
Paroxetine and other anti-depressants	Inhibition of reuptake of serotonine/noradrenaline (SSRI/SNRI)	Antidepressants	Depending on drug in use	Typical side effects of anti-depressants	Krupp, 2003; Zielińska-Nowak et al., 2020; Stamoula et al., 2021
Methylphenidate	Enhancement of dopaminergic effects, CNS stimulants	Improves transmission of dopamine and inhibits its reuptake (typically prescribed for ADHD)	5–20 mg/day (TRIUMPHANT-MS)	Cardiac, psychiatric, gastrointestinal disorders	Nourbakhsh et al., 2018; Cercignani et al., 2021; Tarasiuk et al., 2022
Acetyl-L-Carnitine (ALCAR)	Mitochondrial functionality and ATP production improvement	Increases energy levels based on biochemical production of ATP	2 g/day	Nausea, agitation, insomnia, and increased appetite	Krupp, 2003; Pennisi et al., 2020; Cocco and Fadda, 2022
Pemoline	CNS stimulant	Increases attention and wakefulness (normally used in ADHD)	18.75 mg/day (some studies start from 37.5 mg and decrease to 18.75)	Irritability, restlessness, insomnia, liver function test changes	Krupp, 2003; Braley and Chervin, 2010; Khan et al., 2014; Cocco and Fadda, 2022

* Most commonly prescribed.

#### 2.4.2. Non-pharmacological treatment

##### 2.4.2.1. Supportive strategies

Supportive approaches such as physical rehabilitation, physiotherapy or aerobic exercises, and relaxing sessions (e.g., yoga) have been proven to relieve patients from MS fatigue (Jensen et al., 2014; Picariello et al., 2022; Deutsche Multiple Sklerose Gesellschaft B e. V., 2023). Occupational, cognitive-behavioral, or psychological therapy may be key in the management of fatigue as handling and anticipating triggers to prevent fatigue can be learned (Picariello et al., 2022) as a way to boost self-determination and confidence (Askari et al., 2022).

##### 2.4.2.2. Transcranial stimulation

Among the more innovative treatment strategies, transcranial and non-invasive brain stimulation (NIBS) emerges as an alternative option. Its basic principle is targeting the somatosensory, post-central, and frontal areas with scalp-attached electrodes and low-current stimulation (Ayache and Chalah, 2018; Bertoli et al., 2023). Further, the immediate benefits observed in the QoL of the fatigued MS patients hint at anodal transcranial direct current stimulation (tDCS) as an innovative therapeutic method for fatigue (Ayache and Chalah, 2018; Linnhoff and Heinze, 2019; Bertoli and Tecchio, 2020; Mortezanejad et al., 2020; Zielińska-Nowak et al., 2020). By targeting the primary somatosensory area, Bertoli et al. also managed to lower fatigue levels thus explaining how the mechanism underlying fatigue is rather central than peripheral. Concluding, the main pathophysiological processes take place in neuronal connectivity rather than at a neuromuscular level (Bertoli et al., 2023).

## 3. The concept of digital assessment and management of multiple sclerosis fatigue

As mentioned above, several of the currently used measures for the assessment of fatigue are already profiting from digitalization in data collection, as PROs are easily available, or eHealth approaches make them broadly available. Digital tools are also emerging as diagnostic and therapeutic resources, partially self-supervised or with minimal support from caregivers. As MS patients are often diagnosed at the age of 20–40s, they could be the perfect candidates for the implementation of digital tools in the early phases of their disease. Current generations of newly diagnosed patients are already familiar with digital-based activities. Furthermore, they are highly motivated to find solutions to the limitations MS puts ahead of them (Haase et al., 2021; Scholz et al., 2021).

The use of modern digital solutions might represent an efficient way of sharing information between patients and clinicians, who could thereby build a trusted network with their patients and improve “doctor-patient”-relationships. As data may become easily available, quantifiable, and continuous remote monitoring of fatigue would be possible. These network communications might play an important role for patients who live in underserved areas, where the first healthcare structure is usually distant and difficult to reach. Those patients may especially benefit from long-distance medical care through eHealth (De Angelis et al., 2021; Haase et al., 2021; Ziemssen and Haase, 2021). This was evident during the COVID-19 pandemic as regular outpatient visits were frequently not performable due to the lockdown-related restrictions or their fear of being infected. Therefore, eHealth is proposed as an expedient to bridge the temporal and spatial gap between the visits in the clinics (Haase et al., 2021; Scholz et al., 2021; van der Walt et al., 2021; Voigt et al., 2023).

Voigt et al. have envisioned a way to digitally handle patients through artificial intelligence tools: the concept of the “digital twins” consists of collecting all the data regarding the patients in a digital cloud, which the clinician can easily consult. This enables to monitoring of the disease step-by-step and in an individualized manner so that no detail of acute relapse symptomatology or any kind of worsening condition goes lost or overlooked. At the same time, it is a means to include the patients in the clinical pathway, also as far as medications are concerned, intending to predict the future of this “thousand-faces disease” with a custom-made approach (Voigt et al., 2021, 2023). This all can be achieved thanks to a thorough planning of the resources and a following implementation of apps, wearable devices, machine-learning instruments, and data collection and analysis systems (Dillenseger et al., 2021).

However, patient safety and data protection is also an aspect for consideration in digital tools in MS. Digital applications may be regulated in several countries as they may be considered medical devices (van der Walt et al., 2021). Similar definitions are seen both in the United States, through the Food and Drugs Administration (FDA), and in the European Union, via the Medical Device Regulation (MDR). These are seen simplified as instruments, appliances, software, or other articles intended for medical purposes, including, among others, use in diagnosis, treatment, or prevention (Maaß et al., 2022).

### 3.1. Current implementations in digital fatigue assessment

#### 3.1.1. Apps

Health and medical apps are becoming increasingly available in MS, especially considering, as mentioned above, the young age of MS populations at diagnosis (Zayas-Garcia and Cano-de-la-Cuerda, 2018; Howard et al., 2023). Health apps are “software programs on mobile devices that process health-related data on or for their user” (Maaß et al., 2022). These could be used by every individual, including patients, family, or caregivers. If these applications are used for medical purposes, such as early diagnosis, monitoring, or treatment, they could be considered medical apps and further, medical devices.

In Germany, as an example, medical apps are regulated through the Federal Institute of Drugs and Medical Devices with the definition of Digital Health Applications (DiGAs, in German *digital Gesundheitsanwendungen*). These are HCP-prescribed mobile applications fully financially covered by insurance companies. The German government is still performing thorough licensing processes and studies to regulate DiGAs proposed by different companies, with very restrictive and precise requirements to assure standardized and continuous care, but also personal data protection (Bundesinstitut für Arzneimittel und Medizinprodukte, 2023). This is also the case with several MS-related apps used for the management of fatigue and other clinical complaints. This regulation enables the app owner to feel safe and meanwhile learn content about symptoms and conditions, which are sometimes difficult to access or understand.

The interactive nature of medical apps makes patients feel accompanied in the management of their disease and gives them the advantage of getting notifications and reminders as regards taking medication, asking for prescriptions, and remembering appointments. Financial support from insurance companies makes them an accessible tool for patients, regardless of their financial status or geographical distance from the nearest point of care (Bundesinstitut für Arzneimittel und Medizinprodukte, 2023).

Additionally, clinical experience with these apps is fundamental in the research field, where medical apps can be thoroughly assessed in their advantages and disadvantages, providing collaboration between establishing companies and HCPs (Bundesinstitut für Arzneimittel und Medizinprodukte, 2023). The new era of fatigue treatment begins with its assessment through consistent, standardized, and easy-to-manage resources.

Previously, we discussed the difficulty of building a diagnostic path for fatigue and the utility of fatigue diaries to monitor symptoms, also from the patient's perspective. An automated assessment is in this way a compromise between a steady observation of fatigue and the need for the patient to develop a good insight of this feature of MS. In the following, we discuss apps that demonstrate exemplary practical distance- and home-based evaluation strategies that could also be advantageous for the patient's self-perception and autonomy. Several of these apps have a mixed function including patient education, communication, and administration of medical findings or even neuro-rehabilitation. Currently, further medical apps are in the pipeline for the assessment of fatigue.

A good example of promoting self-tracking is represented by ELEVIDA, a German DiGA established by GAIA (ELEVIDA, 2002; Pöttgen et al., 2018; GAIA AG, 2022; Bundesinstitut für Arzneimittel und Medizinprodukte, 2023). ELEVIDA is specifically designed for MS patients with fatigue in need of special and continuous support. This app involves a digital neuro-rehabilitation (see Table 3) beyond an assessment of fatigue. They can download the app for a limited time based on prescriptions and find exercises, strategies, and virtual dialogues to self-assess and handle fatigue (ELEVIDA, 2002; Pöttgen et al., 2018). It promotes the patient's self-determination and raises awareness on the recognition of one's perception of fatigue, making the experience of e-Health flexible, tailored, and goal-oriented (ELEVIDA, 2002; Dillenseger et al., 2021; GAIA AG, 2022; Stern et al., 2022). The ELEVIDA study carried out by Pöttgen et al. confirmed the beneficial effect of this home-based tool on fatigue levels, with an additional increase in QoL, especially in the cognitive area. A follow-up was also performed, yielding positive results concerning both fatigue and everyday-living efficacy, which must be a primary endpoint in the field of a disabling disease like MS (Pöttgen et al., 2018).

**Table 3:** Summary of the concepts regarding digitalizing MS fatigue assessment and therapy.

**Application**	**Developer**	**Year**	**Requirements**	**Contents**	**Goal**	**Evidence**
Elevida (Gold et al., 2001; ELEVIDA, 2002; Pöttgen et al., 2018; GAIA AG, 2022)	GAIA AG	2018	Smartphone Internet connection	90-days prescription-based program Fatigue specific contents Exercises introduced by videos Interactive journaling	Assessment Management	Decreased fatigue levels as measured by the Chalder Fatigue Scale and FSMC, even after 12 and 24 weeks (follow-up); QoL increased in the fatigue section of the HAQUAMS (Hamburg Quality of Life Questionnaire in Multiple Sclerosis); good effect on ADLs
ElevateMS (Novartis SB, 2017; Pratap et al., 2020)	Novartis Sage Bionetworks	2017	Smartphone Internet connection	Questionnaires Symptom tracking Activity tracking Customizable reminders Clinical-based exercises (e.g., finger-tappingbalance tests, SDMT)	Assessment	High rate of reports on fatigue on the app; data refers to a population located across the US (far-reaching abilities of the app). Increased compliance in MS patients whose link with the clinic has been aided by their HCPs
BRISA App (Fisk et al., 1994a; BRISA, 2022; Mountford et al., 2023)	Temedica GmbH Roche	2022	Smartphone Internet connection	Smiley-based self-assessment and rating of MS symptoms Daily diary	Assessment	High correlation between smiley-based reported fatigue and completion of MFIS (Modified Fatigue Impact Scale) in MS patients, independently of age, sex or time since diagnose
Energize (Babbage et al., 2019; van Kessel et al., 2021)	Duncan Babbage Kirsten van Kessel Paula Kersten	2019	Smartphone (iOS) Internet connection	7-module based course (MS, Beaviour, Thoughts, Emotions, Body, Future and World) Explanatory videos Sleep diary Self-evaluation of fatigue Planning section Quizzes and tests	Assessment Social rehabilitation	Promising results as far as the acceptance by fatigued MS patients (self-management and learning content) although the cognitive effort to complete the units was sometimes high and itself fatiguing
icompanionMS (Van Hecke et al., 2021; Icompanion, 2022)	icometrix	2021	Smartphone Computer Internet connection	Self-tracking Appointment reminder Cognition tests MRI scans upload function HCP portal	Assessment Management	Feasible instrument to assess and monitor fatigue over time (clinically relevant changes as referred to the Neuro-QoL)
icobrainMS (Van Hecke et al., 2021; icometrix, 2023)	Icometrix	2021	Computer Internet connection	Ai software for magnetic resonance analysis	Assessment (not MS specific)	Feasibility of the AI-based MRI reading, high rate of lesion-detection and MS subtypes differentiation
Floodlight (F. Hoffmann-La Roche Ltd., 2021; van der Walt et al., 2021; Roche Fachportal, 2023)	Roche	2021	Smartphone Internet and connection	Motor and cognition functions assessment Shape-drawing (SDMT) Pinching tomatoes (9 HPT) 2-min walk test (Timed-25-Foot Walk Test)	Assessment	Correlation between app-based and clinically administered tests
Samsung gear S2 (Krupp, 1983a, 2003; Abbadessa et al., 2021)	Samsung	2021	Smartphone Smartwatch Internet and Bluetooth connection	Passive and continuous data collection about walking endurance	Assessment	Strong influence of fatigue as measured through the FSS (Fatigue Severity Scale) and the maximum distance walked by MS patients
Fitbit Inspire (Fisk et al., 1994a; Chikersal et al., 2022)	Fitbit Inc.	2021	Smartphone Smartwatch Internet connection	Passive steps, heart rate, sleep tracking	Assessment	Digital phenotyping and passive data collecting to predict fatigue; link between fatigue (according to MFIS, Modified Fatigue Impact Scale) and depression and global MS burden during the COVID-19 pandemic
GENEactive accelerometer (Krupp, 1983a; Guo et al., 2021; Activinsights Ltd., 2023)	Activinsights	2021	Smartphone Smartwatch Internet connection	Passive data on ADLs	Assessment	Digital phenotyping to correlate the ADLs and MS symptoms i.e., fatigue (previously assessed with the FSS and monitored with a fatigue diary); positive correlation between fatigue and depression and poor sleep quality
More Stamina (Krupp, 1983a; Cella and Chalder, 2010; Giunti et al., 2018, 2020; Stamina, 2022)	University of Oulu	2018	Smartphone Internet connection	Self-reported energy estimation and management Gamified collection of points based on energy spent and allocated	Assessment Management	Feasibility, acceptability, and usability studies are ongoing. App-based completion of FSS and Chalder Fatigue Scale and gamification-aided fatigue self-management
Fimo app (Mäcken et al., 2021; Fimo Health GmbH, 2022)	Fimo Health	2021	Smartphone Internet connection	8-week program Learning contents Rehabilitation through yoga sessions, relaxation methods, games and tests for cognition Medication and appointments reminder Social rehabilitation	Management	Proof of feasibility still ongoing
Transcranial direct current stimulation (tDCS) (Krupp, 1983a; Shahid et al., 2011; Charvet et al., 2018; UWCORR, 2020; Bertoli et al., 2023)			Transcranial stimulation kit	Specific targeting of somatosensory and motor cortex with low current stimulation alleviating fatigue levels	Therapy	Significant reduction of fatigue measured through FSS, VAS (Visual Analog Scale) and PROMIS of study (Patient-Reported Outcome Measures Information System) after tDCS (targeting dorsolateral prefrontal cortex); correlation between number of sessions and fatigue levels (most of all for severely fatigued MS patients)
Virtual Reality (VR) (Al-Sharman et al., 2019; Maggio et al., 2019a,b; Cuesta-Gómez et al., 2020; Manuli et al., 2020; Ozkul et al., 2020; Yazgan et al., 2020; Cortés-Pérez et al., 2021; Leonardi et al., 2021; Nascimento et al., 2021; Pagliari et al., 2021; Altunkaya et al., 2022; Casuso-Holgado et al., 2022; Hollywood et al., 2022; Kalron et al., 2022; Moeinzadeh et al., 2022; Dogan et al., 2023; Hsu et al., 2023)			Computer and mouse/controllers (non-immersive VR) Screens, controllers, sensors (semi-immersive VR) Headsets, controllers and/or sensors (immersive VR)	Neuro-rehabilitation through gamification (serious gaming or exergaming) Relaxation exercises Cognition training Motor and balance training Memory and motor memory rehabilitation	Neuro-rehabilitation	Higher reduction of fatigue levels as opposed to conventional therapy or physiotherapy alone, result even more significant when the two are combined. Physical excercise through VR might increase the patients' activity level and therefore lower fatigue ones. Additional increase in compliance through gamification and interaction. Link between improvements in balance and fatigue levels through lower energy outlay.

The ElevateMS app is another tool used to continuously assess MS-related symptoms, including fatigue (Pratap et al., 2020). In its pilot study, 62% of patients reported fatigue, and interestingly, fatigue triggers such as high temperature, stress, and late bedtime could be properly documented. Patients were self-tested not only through questionnaires, but also with more clinical-based evaluations such as the finger-tapping, balance test, and a modified version of the SDMT to assess cognitive information processing speed (Pratap et al., 2020). Objective identification of symptoms and triggers beyond a limited subjective feeling could support the development of future therapeutic strategies. Digital health, however, is continuously changing to adapt to the patient's needs (Voigt et al., 2021). MS patients may have vision disturbances, motility disorders, or hearing problems. These limitations need special consideration in the development of apps or software specifically envisioned for them and therefore tailored to address all the different facets of MS. This is particularly considered in the BRISA app, where the patients can fill out brief questionnaires about the symptoms they can regularly report (i.e., bladder dysfunctions, concentration disorders, or fatigue). Even more interestingly, they can rate their disturbances on a scale from 0 to 4 based on a smiley-face scale (where the 0-smiley represents a poorer state and the 4-smiley a better condition). Apart from that, standardized medical questionnaires (like the Modified Fatigue Impact Scale) are required to be filled out every 2 weeks (Fisk et al., 1994a; BRISA, 2022; Mountford et al., 2023). Although the PROs are still the state-of-the-art method to collect data on the symptoms, this digital solution provides a more immediate and intuitive way to communicate one's symptoms, without having to spend long every day on a retrospective questionnaire. Digital health tools must encounter the patient's need for a quick and steady evaluation method, which leaves no room for interpretation or ambiguity (especially for people with cognitive, linguistic, or visual limitations). Mountford et al. (2023) were able to correlate the daily reported disturbances with the PROs and a rather satisfactory compliance rate, even in the elder patient group.

The Energize app has a more educational function. Through seven sections (i.e., MS, Behavior, Thoughts, Emotions, Body, Future, and World), users learn new concepts and engage in interactive activities (Babbage et al., 2019). The content is displayed through videos and animations (e.g., about fatigue, depression, and rest), whereas MS patients can then report pain, sleep disturbances, deconditioning, and other factors contributing to fatigue. Quizzes are also performed to test the knowledge after the learning phases (Babbage et al., 2019; van Kessel et al., 2021). Apps are also useful for documenting interactions and sharing medical information between HCPs and MS patients. For this purpose, apps such as icompanionMS and icobrainMS were developed (Icompanion, 2022; icometrix, 2023). IcompanionMS is a software in which patients report symptoms, learn strategies to manage fatigue, and share their progress with clinicians. HCPs can view MRIs uploaded by patients on the icobrainMS portal. An artificial intelligence system reads the scans and makes a correlation with functional deficits, such as fatigue, quickly available. The app enables long-distance monitoring and assessment of fatigue symptoms and cognitive disturbances through the Quality of Life in Neurological Disorders (Neuro-QoL), a PRO to assess various domains such as cognition, pain, and social performance in MS (Cella et al., 2012). Regular documentation of symptoms and MRIs, supports the observation of minimal changes in the disease course, although protecting data privacy and without requiring additional hardware. Surveyed patients and HCPs reported positive feedback after using this app as it aided in their therapeutic decision-making (Cella et al., 2012; Medina et al., 2019; Van Hecke et al., 2021; Icompanion, 2022; icometrix, 2023).

Digital monitoring and self-monitoring of MS are also the main focus of the Floodlight MS app (F. Hoffmann-La Roche Ltd., 2021). Floodlight MS focuses on cognition, upper extremity function, and mobility. These are measured through brief exercises (e.g., drawing a shape or matching symbols), balance tests, and walking (Mike Baker and van Band, 2023). In the study carried out by Montalban et al., the tests were successfully correlated with the paper-based examinations used in clinical practice, such as the 9-hole Peg Test, SDMT, and Timed 25-foot walk test (Kellor et al., 1971; Benedict et al., 2006; Motl et al., 2017; Montalban et al., 2022). More conclusive data are collected when other devices are connected to the smartphone containing the app (e.g., smartwatch; see wearables). In this sense, monitoring does not only rely on dedicated tests and questionnaires on smartphones but also on passive monitoring, as highlighted by Montalban et al. (2022).

#### 3.1.2. Wearable devices

Not only apps measuring fatigue through questionnaires but also wearable devices have proved to be useful in the continuous monitoring of fatigued MS patients (Sparaco et al., 2018; Tong et al., 2019; Block et al., 2022). These “health technologies” usually have an internet or smartphone connection and therefore allow non-stop collection of data about physiological parameters (e.g., heart rate, sleep levels) and track any kind of physical activities through accelerometers and sensors (Sparaco et al., 2018).

When routinely worn, wearables can deliver useful information about motor activities, which are strongly influenced by fatigue (e.g., gait, balance, social contacts, sports, etc.). These are non-invasive instruments provided with sensors that can be worn daily. Additionally, the patients can retrieve constant feedback and be aware of performance changes (Alexander et al., 2021). These devices convey a measure of objective fatigue, also known as fatigability, which does not necessarily correlate with the retrospective reports represented by the questionnaires (Linnhoff and Heinze, 2019). Furthermore, objective measures give an insight into state fatigue, that is to say, they precisely quantify its severity in a precise instant (Block et al., 2019). Finding a homogenous system to deliver clinically relevant information about a subjective symptom such as fatigue is necessary. Similarly, an overview of the patient's daily limitations, which are not always possible to objectively observe during the visits, may be relevant in future practice (Block et al., 2022).

Currently, several monitoring systems using data collected through varied wearable devices are becoming available, varying from widely used motion-based models (e.g., acceleration, rate of rotation) to complex indirect reference measures (such as heart rate variability, or even electroencephalography, electromyography, or galvanic skin responses. Machine learning is emerging as a tool for understanding these complex data and its relationship with fatigue. A critical point in the implementation of wearables to detect fatigue is the difficulty of relating it to the triggering task when having the patient keep the wearable outside the hospital or laboratory. In other words, as fatigue is usually provoked by a motor or cognitive activity, not knowing the nature of this activity poses a gap in understanding how and when fatigue caves in. This is the reason why supervised monitoring in the laboratory is still more effective than the long-distance, long-term one. Furthermore, the use of physiological signs to gauge fatigue provides quite an objective mean for real-time monitoring at the individual level. However, individuals have a high variability of responding to stress and fatigue, which translates into a scarce homogeneity of retrieved data. Future works should focus on establishing data-collecting models to better phenotype fatigued MS patients (Adão Martins et al., 2021).

Finding a homogenous system to deliver clinically relevant information about a subjective symptom such as fatigue is necessary. Similarly, an overview of the patient's daily limitations, which are not always possible to objectively observe during the visits, may be relevant in future practice (Block et al., 2022).

The importance of passive data collecting, and wearable devices becomes even more evident when it comes to the correlation between fatigue and other factors influencing the QoL, like ADL, social functioning, but also other comorbidities like depression or sleep disorders. Thereby, sensors can, for example, document the amount and quality of sleep and therefore provide insight into a concomitant factor contributing to fatigue as a PRO cannot assess precisely the scope of this type of disturbances (Bradshaw et al., 2017). The helpfulness of wearables ranges from fall risk management to the gathering of biological digital biomarkers in an uninterrupted period of time, aside from the patient's direct involvement or realization (Vandyk et al., 2022). Another possible advantage is a potential reduction of healthcare costs, as continuous and automatic data generation may represent fewer appointments with the practitioners (Dillenseger et al., 2021).

In a recent study, Abbadessa et al. correlated fatigue PROs with fatigue, depression, and ADL with walking endurance in 25 MS patients. They consequently described the influence of fatigue on the maximum amount of steps per day and the correlation of this objective and passive evaluation with subjective and patient-dependent reports, namely personal evaluations of fatigue and related symptoms (Abbadessa et al., 2021).

In a different approach, Chikersal et al. conducted a study involving a smartphone app and sensor (Fitbit) to check the status of the patients during the COVID-19 lockdown, in which they were homebound and social contacts were reduced. Through comparison between digital biomarkers retrieved by these sensors and the MFIS (completed by the patients every 4 weeks), the burden of fatigue and its worsening through social isolation could be reported. Additionally, a significant overlap with depression was demonstrated (Chikersal et al., 2022).

A similar method involved a fatigue diary to fill every day while at the same time wearing a GENEactive smartwatch to passively record the patients' daily physical biomarkers. The approach established by Guo et al. linked data sampled by the sensors (GENEactive smartwatch) with clinical data from the PROs and daily completion of a fatigue diary. From this 1-week pilot study, a significant relationship between fatigue and depression and sleep quality was objectively assessed. This enabled the authors to build different behavioral phenotypes of MS patients, which present various combinations of different symptoms and therefore require different treatments. A step further has been made in the direction of patient-based care (Guo et al., 2021; Activinsights Ltd., 2023).

In conclusion, the relationship between the completion of the questionnaires and device-reported data specifically concerning fatigue revealed that not only the two kinds of measures can be linked but the technological and objective outcome measure is useful to predict the subjective perception of fatigue. This finding might be a sustainable solution to promote the patient's home-based self-assessment independently from the regular visits but also reinforce the clinician's ability to escort the patients into more conscious joint planning of the long-term disease management (Krupp, 1983a; EQ-5D EG, 2009; Tong et al., 2019; EuroQol Research Foundation, 2021).

### 3.2. Digital neuro-rehabilitation for MS fatigue

Although several studies have examined the use of digital tools for the assessment and treatment of fatigue, especially through mobile apps, clear evidence of digital implementations to target fatigue is relatively scarce. We summarize available evidence for recently developed apps and strategies with virtual reality (VR), as these have been proven to improve the deficits in MS patients. We believe further research may potentially provide insights regarding the use of other digital hardware, such as body scanners, smartwatches, sensors (e.g., gamifying), or wearables in neuro-rehabilitation.

#### 3.2.1. Home-based tools

The crucial focus of developing fatigue-focused software and apps is to demonstrate and strengthen the patients' autonomy. Learning how to recognize the triggering factors and concomitant symptoms with the aid of technological resources is essential to successfully handle or avoid them.

The More Stamina app boosts the patient's self-determination by requiring them to distribute their own resources. MS patients are compelled to autonomously estimate energy levels and motivation to perform given and planned tasks. Based on the activity levels, points can be gathered and through an adaptive interface and game-like design, patients can benefit from positive reinforcement assuring good adherence and participation. They are thereby the active protagonists of their fatigue management through the development of effective energy-distribution strategies (Giunti et al., 2020).

In the Fimo app (Fimo Health GmbH, 2022), home-based digital health support is more steadily conceived: it is an 8-week self-care path, complementary to the one provided by the doctors. It begins with a learning phase about MS and its related fatigue syndrome and then the patients can actively accomplish strategies to overcome it through sports sessions, meditation, relaxation methods, or any kind of action. The cognitive tests provided by this app can be undertaken more often by the patients, who can also report both triggering factors for fatigue and even other kinds of symptoms possibly contributing to a state of discomfort. Besides, the app consists of different steps through which MS patients can finally reconnect to the outside world and have a more efficacious social and work life. As previously mentioned, fatigued MS patients tend to isolate and give up any kind of interaction, fearing the effects fatigue might have on them. An interesting aim of this app is to develop symptom-coping strategies and overcome daily limitations due to fatigue (Mäcken et al., 2021; Fimo Health GmbH, 2022).

Several apps that were primarily developed for the assessment of fatigue in MS patients are also being implemented for neuro-rehabilitation. As mentioned above, the ELEVIDA, Energize, or More Stamina apps could provide innovative management strategies not only for the assessment but also for a self-guided treatment of patients (see Table 3) (ELEVIDA, 2002; Giunti et al., 2018, 2020; Pöttgen et al., 2018; Babbage et al., 2019; van Kessel et al., 2021; Stamina, 2022).

Previously, we discussed the current use of tDCS in the clinical setting. Charvet et al. (2018) discussed in their work the possibility of having it as a home-based neuro-rehabilitation tool. After assessing the disability level through the EDSS and the cognitive functions with the SDMT, participants were treated with a 5-week-long, home-based, HCP-supervised tDCS at home. The target of this stimulation was the dorsolateral prefrontal cortex (DLPFC), a known pathologically relevant area for the onset of fatigue. The study shows how fatigue levels were improved over a short period of time, so it was quickly effective. On top of that, the results proved more significant after 20 sessions compared with the fatigue scores reported by MS patients who received 10 sessions. This lets us hypothesize that tDCS can be a cost-effective and practicable tele-rehabilitation method (Charvet et al., 2018).

Control gained by patients on their condition represents an important step to make them feel in charge of their own situation not only as chronic patients but also as individuals. HCPs are, nevertheless, available and can support them over this monitoring, adding to safety, collaboration, and involvement. The above-mentioned apps can detect daily fluctuations and improvements with the additional benefit of being cost-effective, intuitive in function, and easily updatable and adaptable to the patient's needs and desires (Mäcken et al., 2021).

#### 3.2.2. Virtual reality

VR is a tool for assessing and treating various aspects of MS, including fatigue, as it allows to perform physical but also cognitive rehabilitation, restores neural plasticity, and therefore mends the patients' functional performance, which is severely affected by the pathophysiological damage underlying of fatigue (Leonardi et al., 2021).

On a technical level, non-immersive VR tools comprise task-oriented games without complete isolation, i.e., with computers and a mouse or controllers but no headsets or big screens (Kalron et al., 2022). Semi-immersive VR is achieved through the use of screens of larger dimensions, which usually enable interaction and might also integrate the tracking of movements (Webster et al., 2021; Kalron et al., 2022). Finally, immersive VR is typically delivered through headsets and controllers to perform activities in a fully isolated environment (Cortés-Pérez et al., 2021).

This immersion enhances the patients' involvement, amusement, and finally motivation. This is also valid for the visuo-auditory feedback offered by the system, which is described by Hollywood et al. (2022) as the response to a correctly achieved task by the system itself. Fatigue as a rehabilitation target can be achieved through VR even when the tasks involved include balance and motor performance. Through the head-mounted display, MS patients can only see the virtual surroundings and an avatar reproduction of their body. Without the distractions of the real world and through complete isolation, patients could manage to focus only on the task and restore their memory regarding a specific movement. Importantly, this kind of training must be performed safely in a controlled environment where the patient can be confident that they can move without fear of falling (Ozkul et al., 2020; Yazgan et al., 2020; Hollywood et al., 2022).

The mirror neuron system seems to play a role in this, as the self-representation achieved through VR triggers neuro-plastic connections in the sensory-motor cortical and subcortical areas (Maggio et al., 2019b; Manuli et al., 2020). This provides a prompt and reliable measure of the patient's performance, thus boosting self-awareness and promoting a re-establishment of motor memory. This multisensory experience drives the patient to train further and consequently to gain results from a constant and consistent training program (Maggio et al., 2019b).

Exergaming-based VR, where developing new skills is achieved through actual video games, might be a viable tool to reach the patients' compliance with fatigue-managing programs (Maggio et al., 2019b; Moeinzadeh et al., 2022). Nevertheless, games can also be designed from a rehabilitating perspective and disability-oriented (i.e., serious gaming) (Maggio et al., 2019b). Fatigue plays a role in motor performance and vice versa, in consequently treating or improving the latter aspect, we might retrieve positive results in the former one (Al-Sharman et al., 2019; Yazgan et al., 2020). As observed by Al-Sharman et al., fatigue is a decisive element when planning rehabilitation in the motor domain. By first assessing fatigue and cognition through paper-based tests and then training the motor functions, the impact of the non-motor disability on the physical one was confirmed (Al-Sharman et al., 2019). Ozkul et al. performed an immersive VR-based rehabilitation program targeting balance and mobility but also fatigue. In their study, they used a Microsoft Kinect to collect image analysis data on motion. This data was then handed to the physiotherapist, who adapted the exercise regimen to the patients' capabilities. Lastly, the VR headset presented the virtual environment to the patients, where they carried out the desired task (Ozkul et al., 2020). This example of task- and patient-oriented neuro-rehabilitation method demonstrates how VR can be tailored to the patients.

Pagliari et al. carried out a study involving a VR home-based tele-rehabilitation program accompanied by a clinician's feedback. They witnessed how fatigued MS patients can benefit from VR-based therapy as far as emotional drive is concerned, although they lack the energy to perform simple day-to-day activities. These patients might retrieve so much psychological and physical profit from the therapy that they might eventually go back to work and engage in the social or sports activities they had given up (Pagliari et al., 2021).

A recurring issue in treating MS fatigue is the uneven distribution of resources among the patients, even as far as healthcare structures are concerned. The fact that some of them are already available on the market pictures the future chance to carry out long-distance rehabilitation programs for both upper and lower limbs (Hollywood et al., 2022). An interesting point of view was offered by Manuli et al., who successfully merged digital neuro-rehabilitation through robotics and VR and the Hub-and-Spoke healthcare system, consisting of a central clinic aided by peripheral centers. As a matter of fact, MS patients often require comprehensive and ongoing care. However, when they reside at a significant distance from the clinics where their preferred HCP practice, the implementation of neuro-rehabilitation becomes a challenging endeavor. In this vision, being the peripheral neuro-rehabilitation team trained in the Hub center and always communicating with it, they can provide the patients with continuous care in a place that is closer to their homes (Manuli et al., 2020).

**Figure 2:**
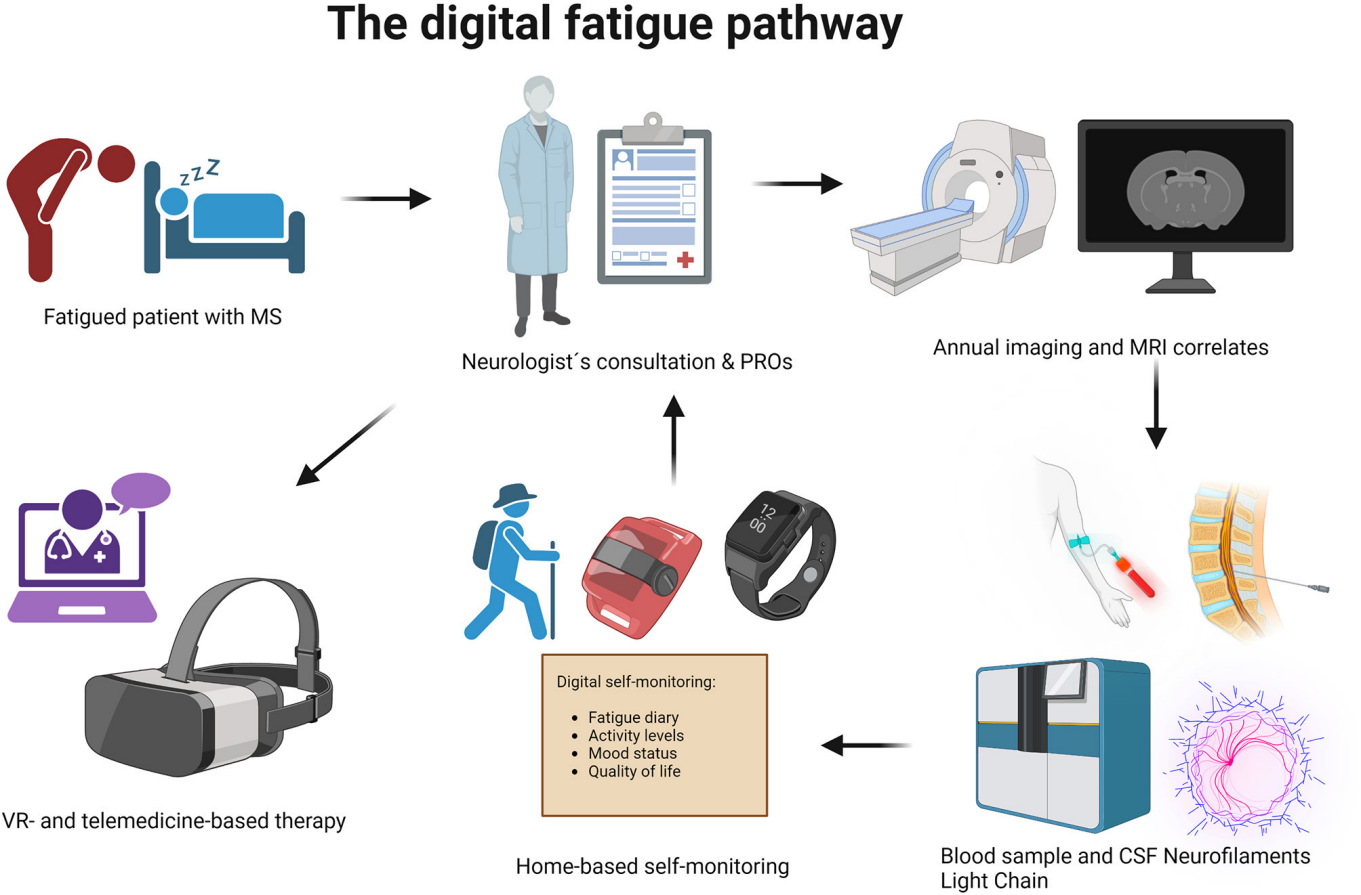
The fatigue path. The clinical pathway usually begins with the MS patient reporting fatigue and exhaustion, which leads them to a consultation with an HCP. In the MS clinical practice, an MRI is to be performed once a year to detect anatomical correlates regarding the clinical evolution of MS. NfL levels are also retrieved in blood and CSF as biomarkers of both physical and cognitive development of MS and fatigue. The observation of fatigue proceeds with the PROs, questionnaires, diaries, and scales. Up until now they have been the most reliable tool to assess fatigue in MS patients. Here, we propose a new way of assessing fatigue in MS patients. With the aid of apps and wearable devices, our clinical pathway model adds a feasible tool to the monitoring of MS fatigue, most of all between visits and at long distances. Finally, neuro-rehabilitation through hospital- and home-based tools and in-clinic virtual reality training can be implemented. Created with BioRender.com.

As a challenging aspect, we must address the dropout rates and compliance of MS patients in VR-based therapy. Some studies have hinted at the fact that a longer duration of the rehabilitation program could cause fatigued patients with MS to lose motivation and thus adherence. Consensus about the right duration of a VR therapy session is one more time difficult to find in the literature, but it is strategic to keep in mind the potential obstacles of VR, which can hinder efficacy and cost-effectiveness (Bevens et al., 2022; Casuso-Holgado et al., 2022). Manuli et al. viewed a possible solution to this gap by adding the patients' reported opinions and outcomes to the concept of feasibility. In their work based on conventional, robotic, and VR-based training, they involved the patients in the evaluation of the activities in terms of usability, perception of the obtained motor, and cognitive goals, and QoL. As these rehabilitation tools are and have to be designed on the patient's baseline disability, training performance, and achievements, this method is a fundamental milestone in the feasibility assessment of these technologies (Manuli et al., 2020). Table 3 summarizes the concepts regarding digitalizing MS fatigue assessment and management and Figure 2 depicts the clinical pathway for fatigued MS patients.

## 4. Discussion and conclusion

Although the anatomical correlates of fatigue in MS patients are not yet fully clear as its subtle nature makes it difficult to evaluate it objectively and to share a common strategy of encompassing it, both in the clinical and the research field. Nevertheless, updated, feasible, and digital are highly promising to make a difference in observing fatigue even at the beginning of its onset. Additionally, the management strategies offered by digital biomarkers, self-assessment instruments, and VR represent the most reliable complementary source of data and treatment outcomes complementary to the HCPs' observation.

As far as the therapeutic use of VR and apps is concerned, published literature is promising but more efforts are required. Nonetheless, in the smoldering frame of MS, where the underlying inflammation plays an equal role as the relapses, the digitalization of neuro-rehabilitation must be addressed as a fundamental element. MS is a complex and multi-faceted disease characterized by thousands of different limitations making the patients different from one another but also differently burdened. The concept of a homogeneous tracking method, as well as a scientific and clinical consensus regarding the healing process, brings us a step closer to changing the face of MS itself. The cost and time effectiveness of the above-discussed tools has been proved by various studies and they could even render a more objective and precise insight into the patient's clinical situation. To reach this goal, eHealth apps and wearable devices deliver a double advantage in assessment: the passive but constant data collection and a successful connection of clinic appointments with HCPs. Furthermore, autonomy could be enhanced as MS patients manage fatigue and learn how to observe and control the triggering factors.

Moreover, VR seems to represent a feasible ally in the field of neuro-rehabilitation. Many studies have highlighted its efficacy in restoring cognitive abilities, thanks to the simulation of motor tasks and simulations of ADLs. For example, Leonardi et al. offered both a neuropsychological evaluation and cognitive training before performing non-immersive VR training with patients. The experimental group improved not only in the motor and cognitive domains but also in the mood, which further demonstrates the effective role of VR in the psychological involvement of MS patients (Leonardi et al., 2021). Additionally, restoring motor memory is a crucial aspect. With standard feedback stimulation, as available through VR devices, this type of memory can be consolidated, along with the awareness of one's motor and cognitive abilities (Manuli et al., 2020). This is especially valid if VR—and gaming-based VR—is administered with conventional therapy. Along with the latter aspect, Leonardi et al., raised awareness of the importance of boosting the cognitive reserve of such patients, as they are very often diagnosed at a younger age. This, together with the increased flexibility and acceptance these younger patients may have toward VR, makes them a target group for a deeper exploration of VR exposure therapy (Leonardi et al., 2021). The concept of ADLs is thereby important: living with MS poses difficulties in day-to-day tasks, ultimately leading to social isolation and even unemployment. Fatigue is in this framework one of the most present symptoms and has therefore to be targeted. By boosting compliance through game-like activities, which request total presence and focus (e.g., immersive VR), we can obtain progress in the memory storing capabilities, motor functions, and QoL outcomes.

Restoring the individual's motor and cognitive functions is not only an integrative approach to be added to the disease-modifying therapies but it represents the therapy itself. As already commented, MS is currently widely considered a “smoldering” and underlying unremitting inflammatory process with superimposed relapses (Giovannoni et al., 2022). This concept paves the way to seeing neuro-rehabilitation as a way to address specific disabilities in order to enhance functional and social capabilities, an outcome which pharmacological therapy has been demonstrated to be lacking to achieve (Maggio et al., 2019b; Giovannoni et al., 2022).

The ultimate stage of this path might be changing the setting of therapy from a hospital- to a home-based one, where MS patients can feel safe and perform tasks with complete autonomy, although always with the support of their HCPs. The next step is to create a safe and secure data-collecting cloud and a trusted relationship between the patients and the new digital implementations, closely followed by a collaboration between patients and clinicians, with the common goal of finding a customized way of care.

## 5. Unmet needs and future perspectives

A controversial topic in the field of digital health and telemedicine is the double requirement of having an individualized pathway to offer to the patients but at the same time providing them with a reliable and standardized assessment. Additionally, data security is an essential point that must not be discounted given that patients and users must have the chance to trust the digital system as they would trust their physician. To achieve this goal, firm protocolling must be made to rule a potential source of both clinical and research advantages but also economic and legislative dark sides e.g., data collection and protection (van der Walt et al., 2021). The collaboration between HCPs, researchers, and developers of digital solutions is crucial to adapt the assessment and the neuro-rehabilitation to MS patients. Similarly, as paper-based methods and symptomatic therapies, digital tools need to reach a high level of reliability to be referred to as “software as medical devices” (van der Walt et al., 2021). The matter of cost and benefits calculations must also be addressed, as it is always a key aspect in the introduction of every new procedure in the medical field. The cost-effectiveness must be advantageous for patients, who are already burdened by the disease and costs related to medical care, and the possible disadvantages of living in a developing country where the technological resources are extremely limited, as well as the digital literacy (De Angelis et al., 2021; Dillenseger et al., 2021). Furthermore, the costs of digital data collection should provide the clinician and the patient with the chance to choose app-based monitoring to aid therapeutic solutions. The same applies to the expenditures in the field of home-based and home-worn devices, which are surely appealing but in many cases also price-intensive (Dillenseger et al., 2021).

Detection of new symptoms must also be punctual and precise, to generate valid data for early identification of disease activity (Cloosterman et al., 2021). Furthermore, digital biomarkers retrieved through the mentioned innovative instruments must allow HCPs to clearly understand them as important parts of the patient's disease history. They must be coded and stored in such a way that is easily accessed and routinely consulted (Voigt et al., 2023).

As far as gaps in the literature are concerned, most studies include almost exclusively female patients, whose EDSS ranges from 0 to 5 or 6, and with RRMS (Ozkul et al., 2020; Nascimento et al., 2021; Moeinzadeh et al., 2022). Unfortunately, most articles failed to describe test batteries or treatment efforts in very highly burdened patients, who might also suffer from fatigue along with all the physical limitations they might present. A future perspective might be a more extensive use of VR in patients with high-grade paralysis or fatigue levels, given the fact that a large amount of VR systems encompass an extreme variety of exercises and tasks (Ozkul et al., 2020).

Another important point in using VR as a therapy is the treatment intensity, duration, and type of tasks. As in every other therapy concept, a standardized length of session must be found for the patient to benefit from it. A final issue to overcome is the precise recruitment of MS patients to collaborate in research and therapeutic pathways, given that not every patient might benefit from these innovative systems, owing to their poor technological ability, older age, or willingness to learn new strategies from scratch (Dillenseger et al., 2021). At the same time, the caregiving and social environment should be considered and actively involved to complement the patient's difficulties in using a new device having disabilities (De Angelis et al., 2021).

All in all, the digital age of fatigue in the field of MS has begun, although with many challenges ahead. Linking standardized assessments and training to customized and goal-oriented individualized remains a central element in the research. Future studies should therefore concentrate their effort on reaching this milestone to routinely implement digital strategies for mitigating and eradicating fatigue in MS patients.

## Author contributions

CP and TZ designed and conceptualized this paper. CP wrote the first draft and organized the methodology under the supervision and with the reviewing of TZ, JE, CB, and HI. All authors contributed to the article and agreed to the submitted version.
